# Qishenyiqi Protects Ligation-Induced Left Ventricular Remodeling by Attenuating Inflammation and Fibrosis *via* STAT3 and NF-κB Signaling Pathway

**DOI:** 10.1371/journal.pone.0104255

**Published:** 2014-08-14

**Authors:** Chun Li, Yong Wang, Qi Qiu, Tianjiao Shi, Yan Wu, Jing Han, Xingyun Chai, Wei Wang

**Affiliations:** 1 Modern Research Center for Traditional Chinese Medicine, Beijing University of Chinese Medicine, Beijing, China; 2 Basic Medical College, Beijing University of Chinese Medicine, Beijing, China; 3 Anzhen Hospital of Capital Medical University, Beijing, China; University of Catania, Italy

## Abstract

**Aim:**

Qi-shen-yi-qi (QSYQ), a formula used for the routine treatment of heart failure (HF) in China, has been demonstrated to improve cardiac function through down-regulating the activation of the Renin-Angiotensin-Aldosterone System (RAAS). However, the mechanisms governing its therapeutic effects are largely unknown. The present study aims to demonstrate that QSYQ treatment can prevent left ventricular remodeling in heart failure by attenuating oxidative stress and inhabiting inflammation.

**Methods:**

Sprague-Dawley (SD) rats were randomly divided into 6 groups: sham group, model group (LAD coronary artery ligation), QSYQ group with high dosage, middle dosage and low dosage (LAD ligation and treated with QSYQ), and captopril group (LAD ligation and treated with captopril as the positive drug). Indicators of fibrosis (Masson, MMPs, and collagens) and inflammation factors were detected 28 days after surgery.

**Results:**

Results of hemodynamic alterations (dp/dt value) in the model group as well as other ventricular remodeling (VR) markers, such as MMP-2, MMP-9, collagen I and III elevated compared with sham group. VR was accompanied by activation of RAAS (angiotensin II and NADPHoxidase). Levels of pro-inflammatory cytokines (TNF-α, IL-6) in myocardial tissue were also up-regulated. Treatment of QSYQ improved cardiac remodeling through counter-acting the aforementioned events. The improvement of QSYQ was accompanied with a restoration of angiotensin II-NADPHoxidase-ROS-MMPs pathways. In addition, “therapeutic” QSYQ administration can reduce both TNF-α-NF-B and IL-6-STAT3 pathways, respectively, which further proves the beneficial effects of QSYQ.

**Conclusions:**

Our study demonstrated that QSYQ protected LAD ligation-induced left VR via attenuating AngII -NADPH oxidase pathway and inhabiting inflammation. These findings provide evidence as to the cardiac protective efficacy of QSYQ to HF and explain the beneficial effects of QSYQ in the clinical application for HF.

## Introduction

Heart failure (HF) is the ultimate consequence of a vast number of cardiovascular diseases. It is one of the leading causes of morbidity and mortality worldwide [1]. It is considered as an progressive yet irreversible process characterized by damaged pump performance and ventricular remodeling (VR) [2]. VR is also believed as the most essential mechanism for an HF occurrence [3]. Therefore, inhabiting VR early is increasingly becoming an effective way to postpone HF induced by myocardial infarction, hypertension and other cardiovascular diseases [4].

Recent clinical studies have indicated that the AngII-induced oxidative stress and inflammation are the critical mechanism of VR. As firstly, the Renin-Angiotensin-Aldosterone System (RAAS) can activate oxidative stress, thus contributing significantly to the deterioration of cardiovascular function, and eventually lead to myocardial remodeling [5]. In this pathway, NADPH oxidase is a crucial factor for bridging RAAS and oxidative stress progress by generating the reactive oxygen species (ROS) in virtual hypertrophy and remodeling of chronic HF. It is also a potential therapeutic target in HF cases [6], [7]. Secondly, HF has been regarded, in the past decades, as a complex cascade of chronic inflammatory reactions that caused gradual cardiac depression [8]. Besides, recent extensive investigations revealed that cytokines, such as tumor necrosis factor-α (TNF-α) and interleukin-6 (IL-6) had important impacts on cardiac outcome as inflammatory markers [9]. Among them, TNF-α has been considered to have detrimental effects on myocardial function by NF-B and eventually result in VR [10]. Numerous studies have also shed light on the role of IL-6 in cardiovascular disease, as long-term IL-6 levels are highly associated with HF by prolongation of STAT signaling, especially STAT3, in cardiacmyocytes [11]. They are also applied as promising molecular targets to treat HF [12].

Traditional Chinese medicine (TCM) has been applied to treat HF for thousands of years, and some herbal formulas have been proven to be effective [13]. Qishenyiqi (QSYQ), one of the most well-known TCM formula, prepared from a composition of six herbs of TCM, including two star herbs, *Radix Astragali mongolici* (‘huang-qi’ in Chinese) and *Salvia miltiorrhizabunge* (‘dan-shen’ in Chinese), and four other adjunctive herbs: *Flos Lonicerae*, *Scrophularia*, *Radix Aconiti Lateralis Preparata*, and *Radix Glycyrrhizae*. This formula is widely manufactured in China in accordance with the China Pharmacopoeia standard of quality control [14], and is commonly applied in routine treatment of HF in China. Randomized, controlled clinical trials proved that it has a definite effect on improving heart function [15].

Our previous study demonstrated that QSYQ can ameliorate myocardial hypertrophy and remodeling by inhabiting the expression of angiotensin II (AngII) in LAD rats [16], improving hemorrheology in HF animals [17], and suppressing angiotensin II receptor levels [18]. However, whether it has protective effect on VR and its underlying mechanisms remained poorly defined. This paper demonstrates that QSYQ has the protective effects against VR in rats. Their targets are related to oxidative stress and inflammation by attenuating STAT3 and NF-κB signaling pathway.

## Materials and Methods

### 1 Animals and grouping

Studies were performed in accordance with the Guide for the Care and Use of Laboratory Animals published by the National Institutes of Health (NIH Publications No. 85-23, revised 1996) and with approval of the Animal Care Committee of Beijing Medical Center. A total of 90 male SD rats in SPF grade were selected (purchased from Beijing Vital River Laboratory Animal Technology Co.Ltd.), weighted 240 g±10 g, and admitted into the study.

### 2 HF Model preparation and study designs

HF was induced by direct coronary ligation as described previously [16]. Before surgery, SD rats were anaesthetized with pentobarbital sodium at a dose of 50 mg/kg. After left thoracotomy and exposure of the heart, the left anterior descending coronary artery (LAD) was ligated with a 5.0 polypropylene suture (Surgipro, CT, USA) directly proximal to its main branching point. Sham groups were made following an identical procedure but without the actual tying of the polypropylene suture. After surgery, as soon as the restoration of spontaneous respiration, the rats were extubated and helped to recover under a heated lamp. They were fed with a standard diet and sufficient water and maintained on a 12 h light and dark cycle.

**Figure 1:**
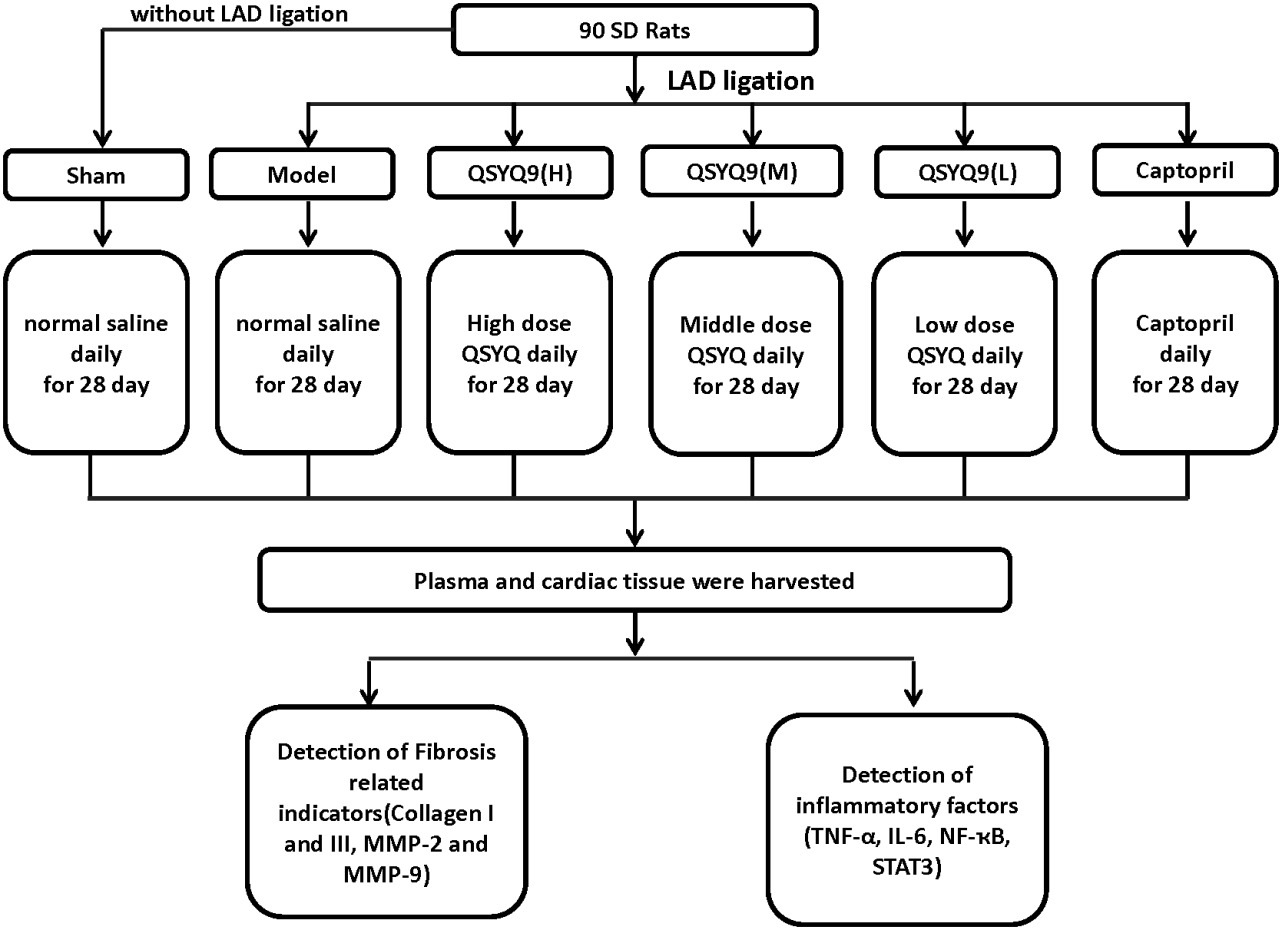
Study designs. Study designs. left anterior descending coronary artery (LAD) was ligated to induce the heart failure (HF) model. Sham groups were made following an identical procedure but without the actual tying of the polypropylene suture. The rats alive after sugery were divided into 5 groups randomly, 14 in model group, 14 in Captopril group and 14 in different dosages of QSYQ groups, respectively. The high dosage QSYQ [QSYQ (H)], middle [QSYQ (M)] and low [QSYQ (L)] groups were administered with QSYQ dissolving in water for 28 days by oral gavage at a total dosages of 9.33 g/kg (H), 4.67 g/kg (M) and 2.33 g/kg (L) of QSYQ on a daily bases. Captopril was given with a dosage of 5.83 mg/kg as a control drug.

The overall mortality rate of model group rats during the entire experiment (28 days after ligation) was 20% to 30%. Among them, 9 rats died of the surgery,16 rats died the day after the surgery, probably because of acute pump failure or lethal arrhythmias. The rats had serious clinical signs, such as short of breath, severe tachycardia. They were euthanized by the injection of 150 mg/kg sodium pentobarbital intraperitoneally. The rats alive were divided into 5 groups randomly, 14 in model group, 14 in Captopril group and 14 in different dosages of QSYQ groups, respectively. Meanwhile, 10 in sham group were investigated together. After the surgeries, all rats were weighed and administered with QSYQ, or normal saline or captopril by gastric perfusion every day for 28 days. The high dosage QSYQ [QSYQ (H)], middle [QSYQ (M)] and low [QSYQ (L)] groups were administered with QSYQ dissolving in water for 28 days by oral gavage at a total dosages of 9.33 g/kg (H), 4.67 g/kg (M) and 2.33 g/kg (L) of QSYQ (Beijing University of Chinese Medicine, Beijing, China) on a daily bases. The dosage choice was based on the recommended daily human dosage and the equivalent conversion between rats and human by body surface area as our previous study [19]. Captopril was given with a dosage of 5.83 mg/kg as a control drug. The sham group and the model groups received the same volume of water via oral gavage as the QSYQ vehicle. At the end of the study, all animals were anaesthetized using isoflurane (AbraxisBioScience, Richmond Hill, Ontario, Canada), followed by an overnight fast. The study designs were carried out as shown in **Figure 1** **.** Cardiac tissue samples were excised parallel to coronary sulcus, 3 mm apart from cardiac apex. All samples were frozen in liquid nitrogen immediately for further examinations.

### 3 Morphometric analysis

Myocardial tissues in the left ventricle (LV) of sacrificed rats (approximately 2 mm in thickness) were removed. Samples were fixed in 4% pre-cooled paraformaldehyde for 72 h and embedded in paraffin for histological studies. Paraffin-embedded tissues were sectioned into slices about 5 µm thicknesses. Masson’s trichrome stain was performed to assess myocardial fibrosis. Images were visualized under an optical microscope at ×400 magnification.

### 4 Hemodynamic measurements in rats

LV performance was measured in rats anesthetized with 2% isoflurane as previously described [20], [21]. A terminal hemodynamic study was performed 4 weeks (28±2 days) after sham operation or LAD ligation. In brief, under anesthesia, right carotid artery, left ventricular systolic and end-diastolic, diastolic and mean aortic pressures, MaxdP/dt, MindP/dt, and heart rate were recorded, using a system of PowerLab ML880 (AD Instrument, Australia).

### 5 Determination of superoxide dismutase (SOD) and malondialdehyde (MDA) by radioimmunoassay (RIA)

The plasma was homogenized in saline containing enzyme inhibitor (0.3 M EDTA-Na 10 uL, 0.34 M 8-hydroxyquinoline 10 uL, 0.32 M dimercaptopropanol 5 ul) (1 ml blood) on ice. The homogenate was centrifuged at 8000×g for 10 min. The supernatant was used for the determination of SOD and MDA using a RIA kit (Beijing Kangyuan Ruide Biotechnology Co. Ltd., Beijing, China) following the instructions of the company.

### 6 Measurement of plasma indicators by ELISA

Levels of TNF-α and IL-6 were quantified by commercial ELISA kits (Crystal Chem Inc., Downer’s Grove, USA). Each assay was performed following related instructions. Standards at a series of concentrations were run in parallel with the samples. The concentrations in the samples were calculated in reference to the corresponding standard curves and were expressed as ng/mL.

### 7 Measurement of indicators by Western blot

The cardiac tissue was homogenized in RIPA buffer (50 mM TrisHCl Ph 7.4, 150 mM NaCl, 2 mM EDTA, 1% NP-40, 0.1% SDS) and total protein was extracted from this homogenate. The protein concentration in each sample extract was measured by a protein assay kit (Pierce Company, USA, lot number: MB155207A) and then was adjusted to the same value in all samples with 2×4% SDS sample buffer. The samples were boiled for 5 mins followed by loading on a 12.5% SDS-PAGE gel (50 mg protein/10 uL per well) for electrophoresis using a Bio-Rad mini gel apparatus at 100 V for 2 h. The fractionated protein on the gel was transferred onto a NC membrane (Beijing PuLilai Gene Technology Co., Ltd, Beijing, China) and electrophoresed at 300 mA for 90 mins. The membrane was first probed with matrix metalloproteinase 2 (MMP2) or MMP9, NADPHoxidase, NF-κB or STAT3 primary antibody (Mouse monoclonal to MMP2, Abcam, USA. Ab3158; Rabbit monoclonal to MMP9, abcam, USA, Ab76003; Rabbit polyclonal to NADPH oxidase, Abcam, USA, Ab60940; Anti-NFκB, Abnova, PAB18492; Anti-NFκB p105/p50 antibody, pNFKB1, Abcam32360, Rabbit polyclonal to STAT3, Abcam, USA. Ab7966) and secondary antibody (donkey polyclonal secondary antibody to rabbit IgG-HRP, ab97064, Abcam, 1∶5000), and then treated with ECL (ECL Plus western blotting detection reagent, GE Healthcare) for 1 min at room temperature. The bands in the membrane were visualized and analyzed by UVP BioImaging systems. After obtaining the MMP-2 (or MMP-9, NADPHoxidase, NF-κB, pNFKB1 or STAT3) blot density, the membrane was then treated by Restore Western Blot Stripping Buffer (Thermo Scientific) to remove the MMP-2 (or MMP-9, NADPHoxidase, NF-κB, pNFKB1 or STAT3) signals, followed by probing with glyceraldehyde-3-phosphate dehydrogenase (GAPDH) primary antibodies (GAPDH mouse monoclonal IgG, Ab8245, Abcam, 1∶2000) using the same process as the MMP-2 antibody to get the MMP-9, NADPHoxidase, NF-κB, pNFKB1, STAT3 and GAPDH blot densities. The final reported data were normalized by GAPDH.

### 8 Measurement of indicators by Immunohistochemistry (IHC)

An avidin-biotin-peroxidase complex commercial method (cell & tissue staining kit, R&D Systems, Inc., USA) was used for IHC. Briefly, 4-mm-thick paraffin wax sections were mounted on slides, which were dried for 30 min in an oven (60–70 centigrade degree) and deparaffinized in xylene. The slides were then placed in changes of ethanol for 2 mins each. Washing in buffer solution was performed between steps. The slides were then placed in 3% hydrogenperoxide for 15 mins and were subsequently incubated in avidin block for 15 mins, biotin block for 15 mins, primary antibody (Anti-Collagen I antibody, Abcam, USA. ab34710; Anti-Collagen III antibody, Abcam, USA, ab7778) for 12 h at 4 centigrade degree, and biotinylated secondary antibody for 1 hour. The reagent incubation was performed with streptavidin peroxidase for 15 mins. A 1 min Mayer’s hematoxylin counter stain was used. The slides were dehydrated, cleared with xylene, and mounted with permanent mounting medium. Finally pictures were analyzed by IPP 6.0 software.

### 9 Statistical analysis

All data were presented as mean ± standard deviation (SD). Statistical analysis was carried out on three or more groups by one-way analysis of variance (ANOVA) and Dunnetts’ test. The values of *p*<0.05 were considered statistically significant.

## Results

### 1 QSYQ treatment attenuated LAD-induced hemodynamic alterations

HF model group was associated with impaired diastolic and systolic LV functions, which were largely attenuated by the treatment with QSYQ for 28 d. As shown in **Table 1**, the indicators reflecting the LV systolic function including LV systolic pressure (LVSP) and Max dP/dt reduced by 23.14% and 34.11% respectively. When treated by different dosages of QSYQ, the values of two indicators were notably recovered (*p*<0.05–0.01). Compared to model group, the captopril group also exhibited a significant promoting effect on LVSP and Max dP/dt (*p*<0.05–0.01).

**Table 1:** Hemodynamic alterations in different groups (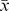
± s).

Group	N	LVSP (mmHg)	LVEDP (mmHg)	HR (BPM)	MaxdP/dt(mmHg/s)	MindP/dt(mmHg/s)
**Sham**	10/10	211.90±20.032**	−28.36±12.129**	416.28±63.630	8389.01±1174.404**	−5362.59±978.656**
**Model**	14/14	162.87±8.997▴▴	−5.23±20.884▴▴	393.08±137.573	5527.27±703.294▴▴	−3511.02±403.734▴▴
**QSYQ (H)**	14/14	190.72±16.039*	−27.15±5.770**	404.41±62.745	7687.42±1162.700**	−4671.15±644.929*
**QSYQ (M)**	14/14	192.50±24.563*	−20.98±11.578*	416.78±69.329	7468.21±1297.474**	−4330.94±612.768
**QSYQ (L)**	14/14	190.03±22.892*	−23.95±11.442*	394.02±63.153	7450.21±1640.391**	−4493.49±1003.122*
**Captopril**	14/14	201.19±13.999**	−26.60±7.632**	418.13±39.357	7976.97±746.919**	−4563.19±1169.573*

▴▴ *p*<0.01, vs sham group, vs sham group;

* *p*<0.05, vs model group,

** *p*<0.01, vs model group.

LV end-diastolic pressure (LVEDP) and Min dP/dt, which were associated with the LV diastolic function, showed a reverse changes. Compared with sham group, LVEDP in model group increased by 81.56%, Min dP/dt was also up-regulated by 34.53%, suggesting an injured diastolic function in model rats. QSYQ at dosage of 2.33 mg/kg, 4.67 mg/kg, and 9.33 mg/kg orally for 28 d could reduce LVEDP and Min dP/dt in different degree compared to model rats (*p*<0.05–0.01). The results showed protective effects of QSYQ on improving cardiac function. Captopril can also play the same action on these indicators (*p*<0.05–0.01). No difference was observed in heart rate (HR) among different groups.

### 2 Effects of QSYQ on MASSON

**Figure 2:**
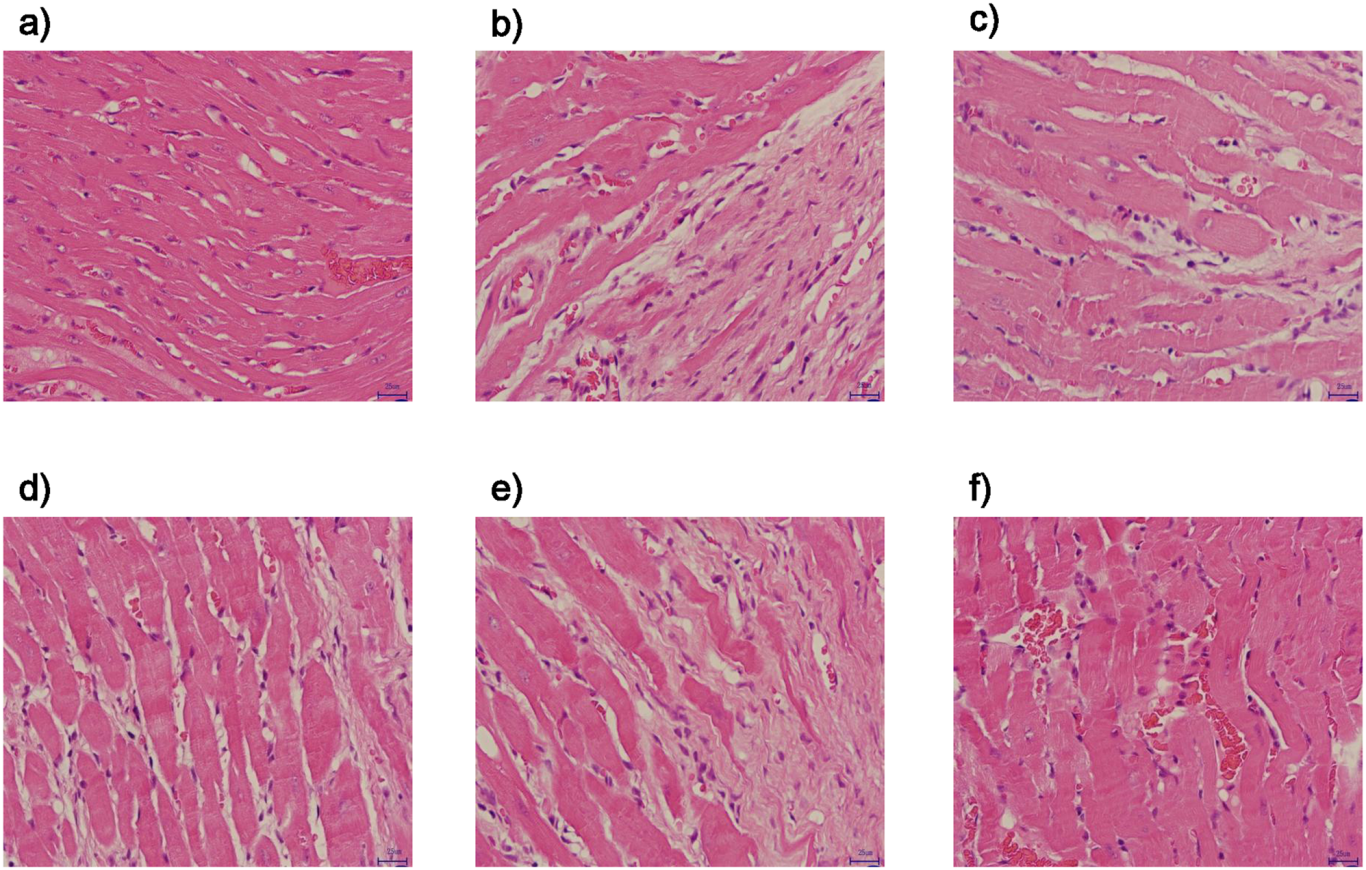
Effects of QSYQ on HE results after occlusion of the left anterior descending (LAD) artery in rats. Cardiomyocytes in the sham group were orderly arranged, and the nuclei were lightly stained and located in the center of muscle fibers. Thickening and lengthening of myocardial fibers could be observed in model group, wherein the nuclei were darkly stained. Cardiomyocyte hypertrophy, cellular degeneration and inflammatory cell infiltration were significantly improved in the different dosage of QSYQ group and Captopril group by contrast with those in the model group. a). cardiomyocytes in sham group; b). cardiomyocytes in model group; c) cardiomyocytes in QSYQ (H); d) cardiomyocytes in QSYQ (M); e) cardiomyocytes in QSYQ (L); f) cardiomyocytes in Captopril group.

The HE-stained images of left ventricular tissue were shown in **Figures 2** **and** **3**, where cardiomyocytes in the sham group were orderly arranged, and the nuclei were lightly stained and located in the center of muscle fibers. Thickening and lengthening of myocardial fibers could be observed in model group, wherein the nuclei were darkly stained, and showing local tissue fibrosis. Cardiomyocyte hypertrophy, cellular degeneration and inflammatory cell infiltration were significantly improved in the different dosages of QSYQ groups and captopril group by contrast with those in the model group.

**Figure 3:**
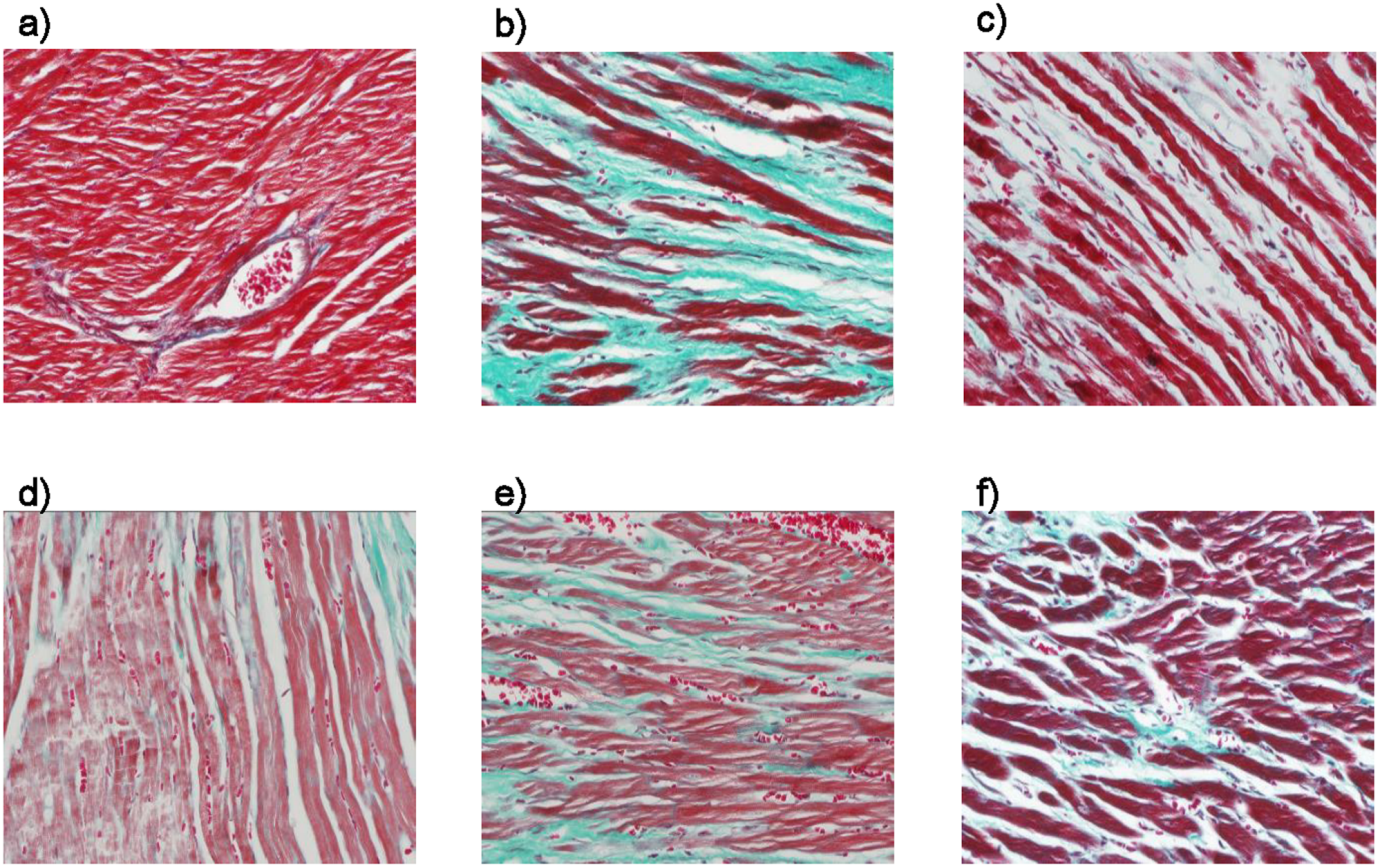
Effects of QSYQ on MASSON results after occlusion of the left anterior descending (LAD) artery in rats. Representative results of Masson trichrome staining of the left ventrentricle in sham group (a).; model group (b).; QSYQ (H) (c).; QSYQ (M) (d).; QSYQ (L) (e).; Captopril group (f). Blue staining indicates myocardial fibrosis. Masson trichrome staining revealed different degree fibrosis in different groups. In sham group, there were almost no fibrosis region. While in model group, there were significant fibrosis region. the increase was significantly attenuated in different dosage of QSYQ groups and Captopril group by contrast with those in the model group. n = 10∼14 for each group.

### 3 Effects of QSYQ on collagens I and II

**Figure 4:**
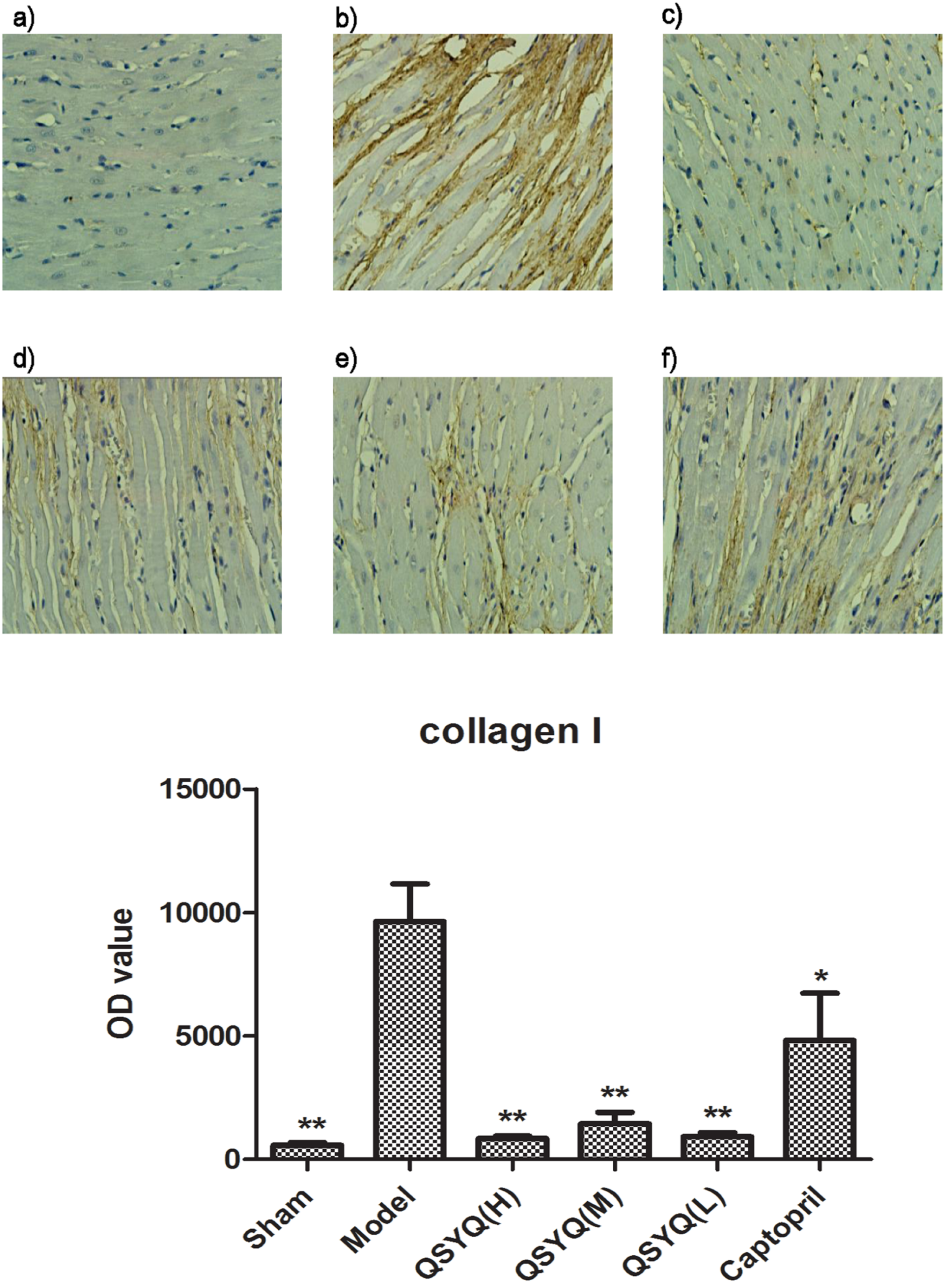
Results of Collagen I expression in different groups. The concentrations of collagens I in the model group (b) were obviously higher than those in sham group (a), indicating a cardiac remodeling in model rats. Whereas the levels of collagens I in high QSYQ (c), middle QSYQ (d) and low QSYQ groups (e) were remarkably lower than those in the model group. Captopril group (f) showed a same effect on suppressing the collagen synthesis. n = 10∼14 for each group. Quantitative analysis of collegen I was presented in (g). OD values represent the mean ± SD. *P<0.05 vs Model group, **P<0.01 vs Model group.

The levels of collagens I and III were detected by IHC and the results were presented in **Figures 4** **–** **5**. The levels of collagens I and III in the model group were obviously higher than those in sham group, with an increasing of 1589.78% and 3359.57%, respectively, indicating a cardiac remodeling in model rats. Whereas the levels of these markers in different QSYQ groups were remarkably lower than those in the model group. Especially in collagen III, there is a dose-dependent manner in reduction rates. The captopril group showed the same effect on collagen synthesis (*p*<0.01).

**Figure 5:**
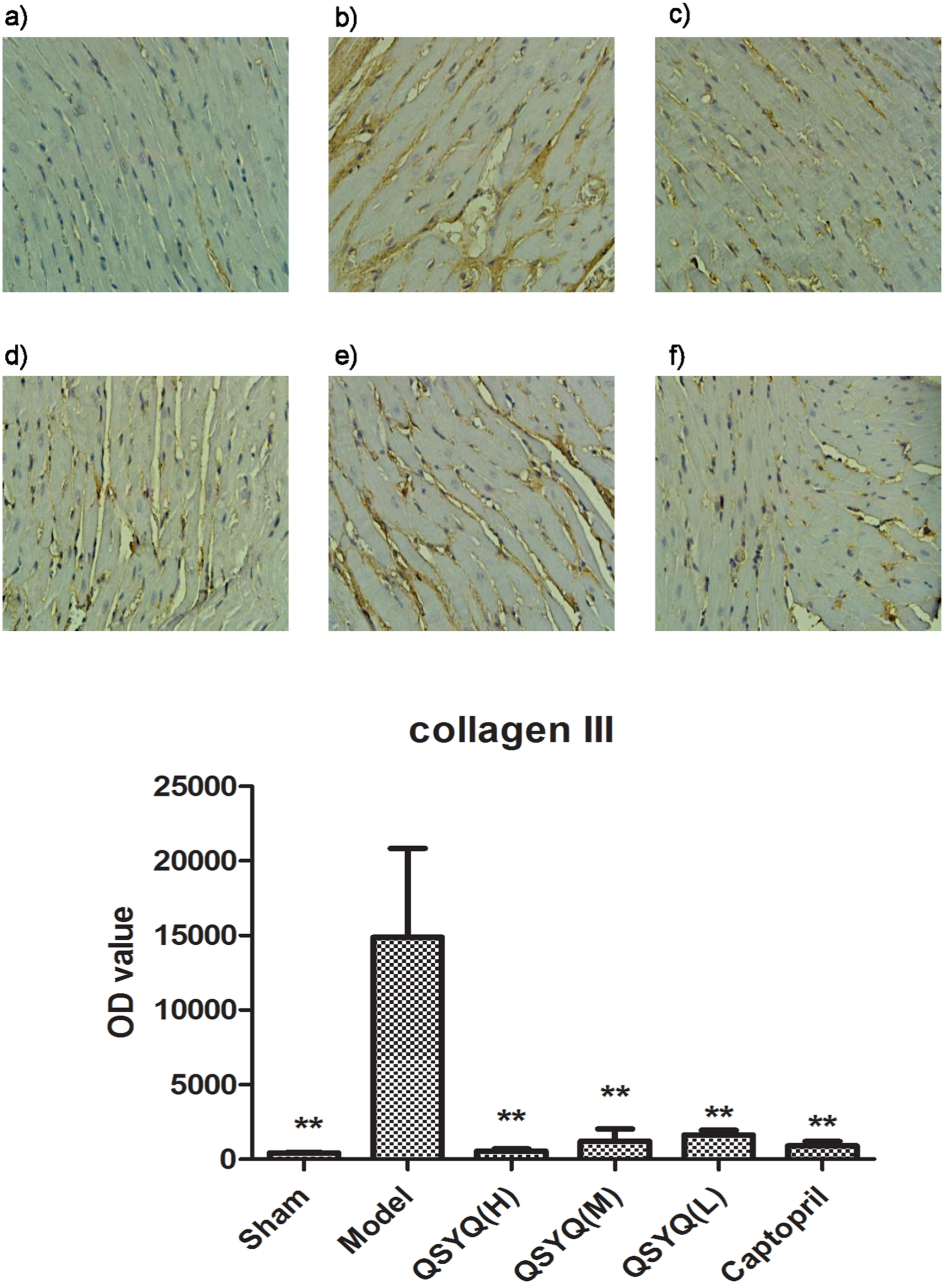
Results of Collagen III expression in different groups. The concentrations of collagens III in the model group (b) were obviously higher than those in sham group (a). Whereas the levels of collagens III in high QSYQ (c), middle QSYQ (d) and low QSYQ groups (e) were remarkably lower than that in the model group. There is a dosage-dependent manner. Captopril group (f) showed a same effect as QSYQ. Quantitative analysis of collegen III was presented in (g). n = 10∼14 for each group. OD values represent the mean ± SD. *P<0.05 vsModel group, **P<0.01 vs Model group.

**Figure 6:**
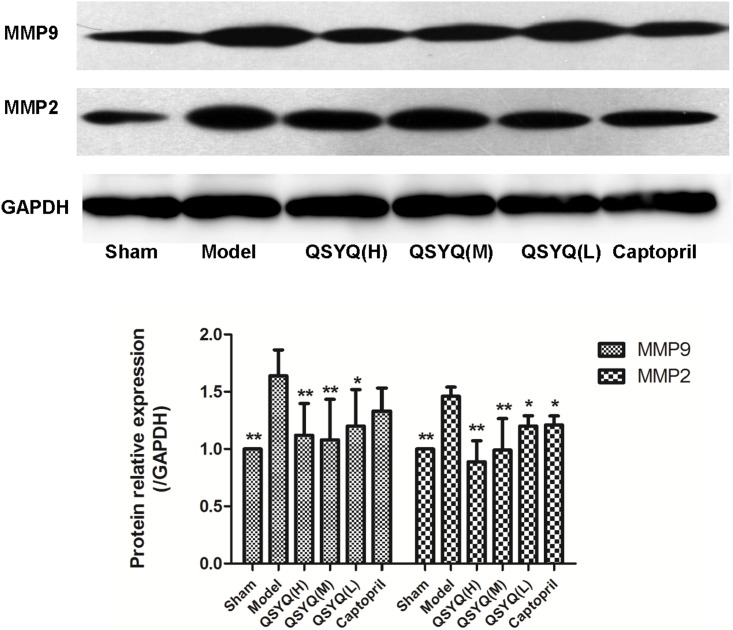
QSYQ treatment significantly decreased cardiac MMP-2 and MMP-9 in rats with HF. MMP-2 and MMP-9 were remarkably up-regulated in model group, indicating elevated degree of remodeling changes. QSYQ (H) greatly suppressed myocardial hypertrophy and remodeling by MMP-2 and MMP-9. QSYQ (M) and QSYQ (L) can also play the same effect. The captopril only suppressed expression of MMP-2, without significant effect on MMP-9. n = 10∼14 for each group. Data were analyzed by one-way ANOVA, with p<0.05 indicating statistical significance. *p<0.05 and **p<0.01 vs. model group.

To further confirm myocardial hypertrophy, two major indicators of LV remodeling, proteases MMP-2 and MMP-9, were detected by Western blot respectively. As revealed in **Figure 6** **.** Compared with sham (1.00±0.000), MMP-2 (1.46±0.080) and MMP-9 (1.64±0.226) were remarkably up-regulated in model group. Combined with IHC, the results indicated LV remodeling occurred in model rats. QSYQ (H) greatly suppressed myocardial hypertrophy and remodeling by MMP-2 (0.89±0.182) and MMP-9 (1.12±0.227). QSYQ (M) and QSYQ (L) also played the same effect on MMP-2 and MMP-9. Captopril only suppressed expression of MMP-2, but had no significant effect on MMP-9. Therefore, the results demonstrated that QSYQ could definitely inhibit myocardial remodeling in HF.

### 4 Effects of QSYQ on oxidative stress

**Figure 7:**
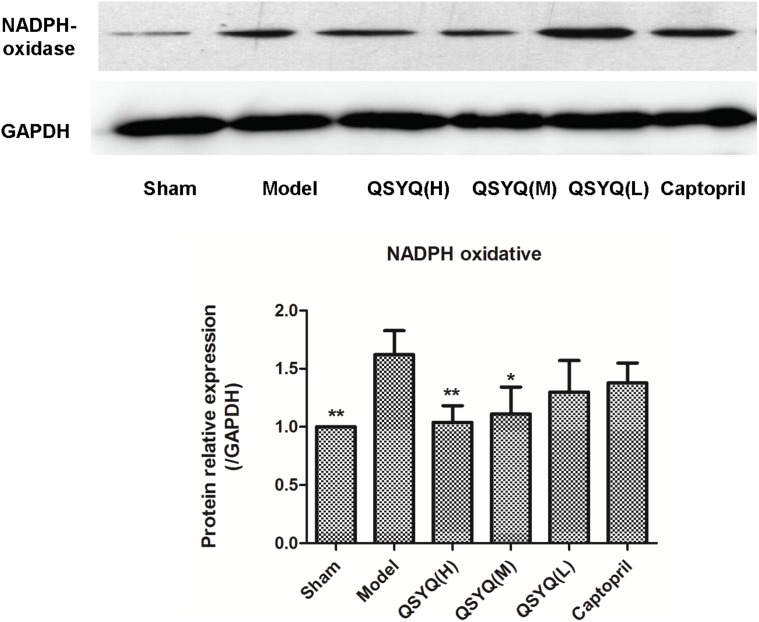
QSYQ treatment significantly decreased cardiac NADPHoxidase in rats with HF. The NADPHoxidase in model group was increased compared with sham group, while treated by QSYQ (H) and (M) doses, the levels showed 35.80% and 31.48% reduction, respectively. The treatment with QSYQ in low-dosage and captopril didn’t improve the NADPHoxidase significantly. The effect of QSYQ on NADPHoxidase showed a dosage-dependent manner. n = 10∼14 for each group. Data were analyzed by one-way ANOVA, with p<0.05 indicating statistical significance. *Differed significantly from the model group (p<0.05).

Oxidative stress was evaluated by detection of MDA, SOD in plasma, and NADPHoxidase in the supernatant of cardiac tissue. As shown in **Table 2** and **Figure 7** **,** a significant increase on MDA level and decreasing on SOD activities in the model group were observed compared to the sham group. QSYQ (H) and (M) could significantly increase the levels of SOD (148.70±32.041, 134.29±20.423) (*p*<0.01), respectively, and decrease the levels of MDA (5.24±1.736 nd 5.13±1.044) respectively (*p*<0.05) (**Table 2**). QSYQ (L) and captopril had no significant effects on SOD, but both can significantly decreased the MDA level.

**Table 2:** Assessment of oxidative stress in different groups (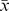
± s).

Group	N	SOD (U/mgprot)	MDA (nmol/mgprot)
**Sham**	10/10	145.87±14.807**	5.74±0.992*
**Model**	14/14	97.94±3.388^▴▴^	7.67±0.896^▴^
**QSYQ (H)**	14/14	148.70±32.041**	5.24±1.736*
**QSYQ (M)**	14/14	134.29±20.423**	5.13±1.044*
**QSYQ (L)**	14/14	117.62±22.356	5.32±1.274*
**Captopril**	14/14	111.17±19.954	5.50±0.943*

vs: compared with model, *p<0.05, **p<0.01; compared with sham, ^▴^p<0.05, ^▴▴^p<0.01.

The Western blot results showed that the NADPHoxidase in model group (1.62±0.208) was increased by 62.40% (*p*<0.05) compared with sham group (1.00±0.000). While treated by QSYQ (H) and (M) doses, the NADPHoxidase level (1.04±0.142; 1.11±0.232) showed 35.80% and 31.48% reduction, respectively, compared to model group (*p*<0.05–0.01) (**Figure 7**). Low doses of QSYQ and captopril did not increase NADPH oxidase expression. Thus, the effect of QSYQ on NADPH oxidase expression was dose-dependent.

### 5 QSYQ attenuated NF-κB activation and inflammation

The ELISA results showed that TNF-*α* and IL-6 were predominately up-regulated in model group by 38.78% and 60.30%, respectively. QSYQ (H) and (M) groups effectively down-regulated TNF-*α* by 13.98% and 21.32%, respectively. Meanwhile, they also could down-regulate IL-6 by 54.37% and 45.04% respectively. These results demonstrated a notable anti-inflammation efficacy of QSYQ. QSYQ (L) and captopril only reduced the expression of IL-6 (*p*<0.05), but had no significant effect on TNF-α **(** **Table 3** **)**.

**Table 3:** Levels of inflammation indicators in different groups (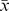
± s).

Group	N	IL-6 (pg/ml)	TNF-α (ng/ml)
**Sham**	10/10	218.08±65.418**	2.13±0.153**
**Model**	14/14	549.45±126.580^▴▴^	2.86±0.304^▴▴^
**QSYQ (H)**	14/14	250.70±65.140**	2.46±0.266*
**QSYQ (M)**	14/14	301.93±112.524**	2.25±0.419**
**QSYQ (L)**	14/14	346.67±32.633**	2.59±0.280
**Captopril**	14/14	318.32±70.427**	2.58±0.328

VS: compared with model, * *p*<0.05, ** *p*<0.01; Compared with sham, ^▴▴^ *p*<0.01.

**Figure 8:**
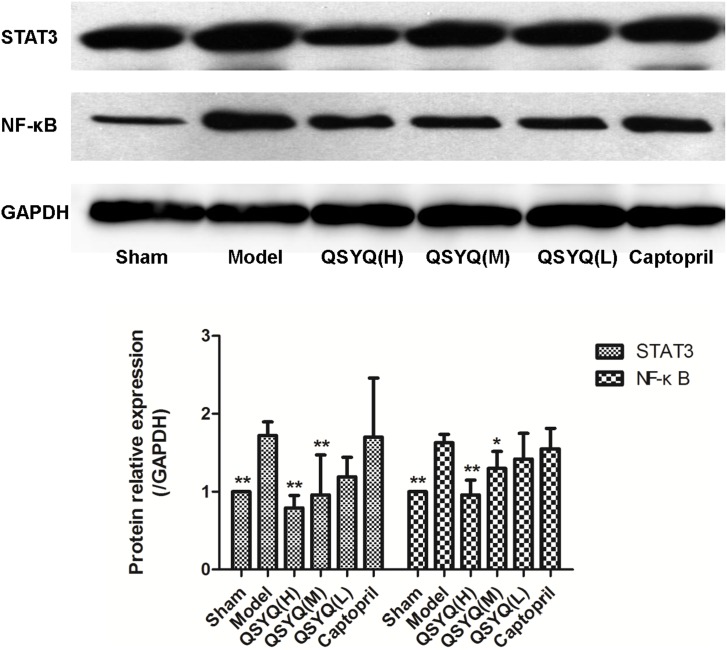
QSYQ significantly decreased cell signal transduction pathway factors of STAT3 and NF-κB in rats with HF. NF-κB in model group increased compared with sham, while treated by QSYQ (H) and (M) doses, the NF-κB level was significantly decreased. TheSTAT3 level in model group was up-regulated, while QSYQ at doses of 9.33 mg/kg (H) and 4.67 mg/kg (M) significantly decreased the STAT3 level respectively. After the administration of QSYQ (L) and captopril, the level of NF-κB and STAT3 had no statistical significance compared to model group. n = 10∼14 for each group. Data were analyzed by one-way ANOVA, with p<0.05 indicating statistical significance. *Differed significantly from the model group (p<0.05).

**Figure 9:**
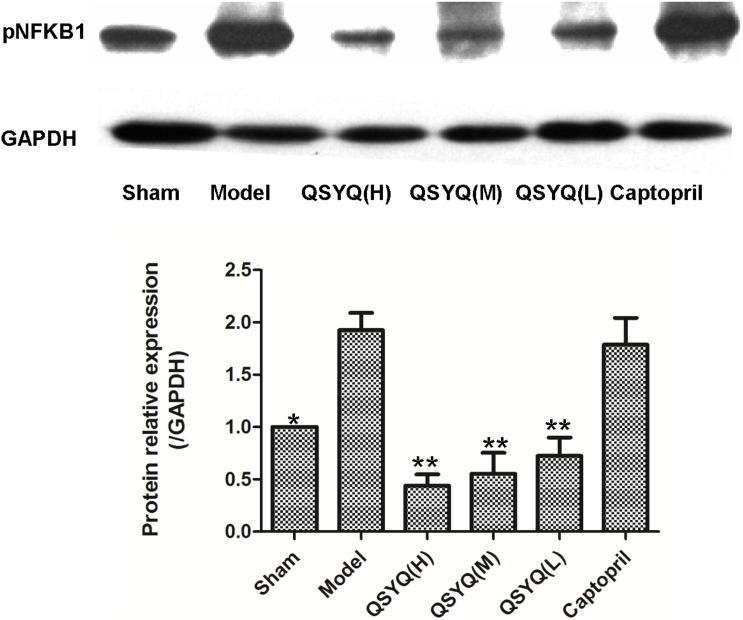
QSYQ significantly decreased cell signal transduction pathway factors of pNFKB1 in rats with HF. pNFKB1 in model group increased compared with sham, while treated by QSYQ (H), (M) and (L) doses, the pNFKB1 level was significantly decreased. In captopril group, the level of pNFKB1 had no statistical significance compared to model group. n = 10∼14 for each group. Data were analyzed by one-way ANOVA, with p<0.05 indicating statistical significance. *Differed significantly from the model group (p<0.05), **Differed significantly from the model group (p<0.01).

Moreover, the pathway protein NF-κB and pNFKB1 was also investigated. It’s a critical signaling molecule which can mediate TNF-α producing apoptosis and oxidative stress. The NF-κB level in model group (1.63±0.107) increased by 63.41% (*p*<0.05) compared with sham group (1.00±0.000). QSYQ (H) and (M) dosages significantly decreased the NF-κB level to 0.96±0.190 and 1.30±0.217 (*p*<0.05–0.01), respectively. Surprisingly, the changes of pNFKB1 in different groups were more significant than NFκB. The results showed that in model group, the pNFKB1 were greatly activated (1.93±0.164) than in the sham group (1.00±0.000). QSYQ in different dosages can down-regulated the pNFKB1 in dose-dependent manner (0.438±0.109; 0.553±0.201; 0.724±0.176 respectively), which were consistent with the change of NF-κB. Captopril had no effect on the pNFKB1. (**Figure 8** **and** **Figure 9**).

STAT3 mediated by IL-6 is another major cause to cardiac hypertrophy [22]. Interestingly, our present data displayed that theSTAT3 level in model group (1.72±0.177) was up-regulated by 71.71% (*p*<0.05) compared with sham (1.00±0.000). QSYQ (H) and (M) significantly decreased the STAT3 level to 0.79±0.161 and 0.96±0.512, respectively (*p*<0.01). (**Figure 8**).

After the administration of QSYQ (L) and captopril, the level of NF-κB and STAT3 had no statistical significance compared to model group (P>0.05). As the results shown, QSYQ not only could improve the VR, but also evidently has a better efficacy on the anti-inflammation than captopril by depressing the TNF-α and IL-6.

## Discussion

Accumulating evidence suggest that the increased oxidative stress coupled with activation of various downstream pro-inflammatory and cell death pathways play pivotal role on the development of complex alterations associated with HF [23]. However, despite of the accumulating knowledge in the past decades, the treatment of HF still remains poor and largely symptomatic [24].

Our research evaluated the effects of QSYQ treatment on myocardial dysfunction, inflammation, oxidative stress, and inter-related signaling pathways, using a rat model of LAD-induced HF. Since significant cardiac dysfunction as well as oxidative stress in this model occurs soon after the operation, with gradually increasing fibrosis and inflammation thereafter [25]. In the treatment protocol, we aimed to explore whether QSYQ can prevent the development of LAD ligation-induced left VR *via* attenuating oxidative stress and inhabiting inflammation.

Consistently with previous reports, the model group animals were characterized by declined diastolic and systolic myocardial performance such as hemodynamic alterations of LVSP, LVEDP, Max dP/dt, Min dP/dt, attenuated antioxidant defense (decreased SOD activity) coupled with increased myocardial ROS generation (up-regulation of MDA and NADPHoxidase). Our results were also in agreement with previous studies, demonstrating the enhanced activation of cardiac inflammatory markers, such as TNF-α and IL-6 and collagen contents associated with HF [10]. The LAD induced-ROS generation also activates pro-inflammatory pathways molecules such as NF-κB [26] and STAT3 [27], [28], which reinforce, in turn, the expression of remodeling markers MMP-2 and MMP-9. QSYQ treatment was able to attenuate the oxidative stress and alterations of the above mentioned cardiac remodeling indicators (collagens I and III) as well as MMP-2 and MMP-9. Interestingly, it also depresses the activations of both TNFα-NF-κB and IL-6-STAT3 pathways. The beneficial effects of QSYQ could be explained, in part, by its potential anti-inflammatory properties. Our recent results suggested that QSYQ may exert potent effects on key pro-inflammatory cytokines, including IL-1 and C-reactive protein (CRP) *in vivo*. These results are also in support of the emerging role of the inflammation in the development and progression of HF [29], [30].

**Figure 10:**
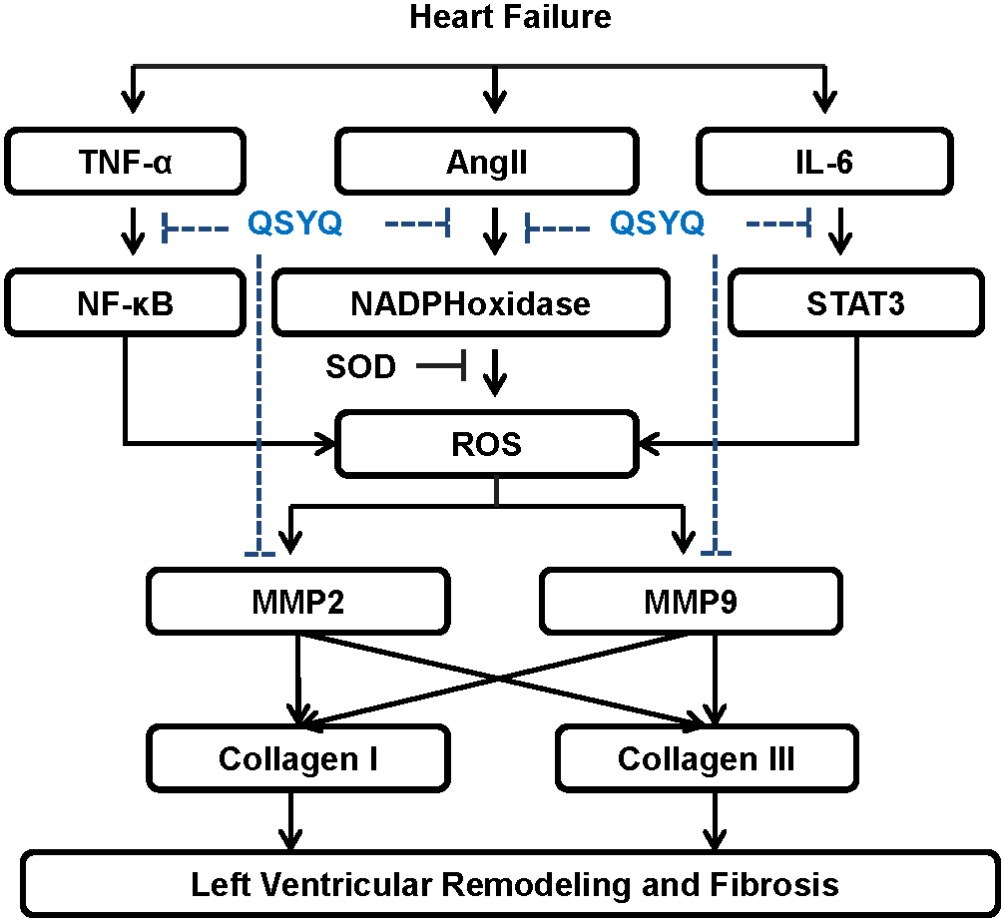
Protocol of the underlying mechanism of QSYQ in attenuating oxidative stress and inflammation signaling pathways in HF rats. QSYQ can reduce Left Ventricular Remodeling by Attenuating Inflammation and NADPH oxidase. It down-regulated the TNFα- NF-B and IL-6- STAT3 respectively to reduce the inflammation in heart failure. Futhermore, it also can supress the NADPH oxidase mediated oxidative stress injury to myocardial tissue. Finally, QSYQ can reduce the MMPs and collegen I and III in different degree and finally provide beneficial effects for clinical heart failure.

The present study demonstrates that QSYQ, an ancient formula composed of six TCM, including two major herbs: Radix Astragali mongolici and Salvia miltiorrhiza Bunge, and four other adjunctive herbs, in a certain ratio, attenuate the cardiac remodeling by checking the MASSON and collagens I and III in HF rats. As shown in **Figure 10**, its cardioprotective efficacy is a comprehensive results by different pathway in a synergistic manner, including restoration of Ang II-NADPHoxidase-ROS-MMPs pathways and reduction of both TNF-α-NF-κB and IL-6-STAT3 pathways, thus to restore the hemodynamic parameters, normalize the cardiac function, and provide the comprehensive cardiac protective efficacy to HF.
